# Multifocal Inflammatory Pseudotumor Involving the Orbit, Skull Base, and Cauda Equina Causing Progressive Visual Loss in a Patient With HIV Infection: A Case Report

**DOI:** 10.7759/cureus.116813

**Published:** 2026-09-24

**Authors:** Kenji Tokunaga, Hirotomo Nakata, Jun-Ichirou Yasunaga

**Affiliations:** 1 Department of Hematology, Rheumatology and Infectious Diseases, Kumamoto University Hospital, Kumamoto, JPN

**Keywords:** central nervous system, covid-19, hiv infection, inflammatory pseudotumor, visual impairment

## Abstract

Inflammatory pseudotumor (IPT) is a rare benign inflammatory mass lesion characterized by inflammatory cell infiltration and fibroblastic proliferation. Although IPT can occur in various organs, central nervous system (CNS) involvement is rare. We report a case of multifocal IPT involving the orbit, skull base, and cauda equina in a patient with a well-controlled HIV infection. A man in his 50s with a 13-year history of HIV infection presented with progressive visual impairment. His HIV viral load remained undetectable under antiretroviral therapy, with CD4-positive lymphocyte counts consistently above 400/μL. Serial magnetic resonance imaging demonstrated enlargement of the skull base and orbital lesions over two months, together with the appearance of additional lesions involving the optic chiasm, pituitary region, cervical spinal canal, and cauda equina. Cerebrospinal fluid examination revealed lymphocytic pleocytosis, without evidence of malignancy or infection. Biopsies of the lacrimal gland and cauda equina revealed inflammatory lymphocytic infiltration without evidence of lymphoma, IgG4-related disease, or other neoplastic disorders. Based on the clinicopathological and radiological findings, a diagnosis of multifocal IPT was established. No corticosteroid or disease-specific therapy was administered before histopathological confirmation because the diagnosis remained uncertain. Following the diagnosis of multifocal IPT, the lesions stabilized without specific treatment despite severe visual loss. This case highlights IPT as a rare differential diagnosis of multifocal CNS lesions in patients with HIV infection, even in the setting of sustained virological suppression and immune reconstitution. Rapid disease progression was temporally associated with recent COVID-19 infection; however, a causal relationship could not be established.

## Introduction

Inflammatory pseudotumor (IPT) is a rare, non-neoplastic inflammatory lesion characterized by the infiltration of inflammatory cells and fibroblastic proliferation. Although the lung and orbit are the most frequently affected sites, IPT has been reported in almost every organ system, including the central nervous system (CNS). A recent systematic review confirmed that CNS involvement is uncommon and presents substantial diagnostic challenges because its clinical and radiological features often mimic those of neoplastic and infectious diseases [1]. Because its clinical, radiological, and histopathological features often overlap with those of neoplastic and infectious disorders, the diagnosis of IPT is established primarily by exclusion of these mimics. The pathogenesis of IPT is incompletely understood, and infectious, autoimmune, and immune-mediated mechanisms have been implicated [2,3]. In patients with HIV infection, virus-associated IPT or IPT-like lesions have also been reported, including cases associated with Epstein-Barr virus (EBV), cytomegalovirus (CMV), and John Cunningham virus (JCV) [4-6].

Several disorders closely mimic IPT, including inflammatory myofibroblastic tumors (IMT), IgG4-related disease, malignant lymphoma, and EBV-associated IPT-like follicular dendritic cell sarcoma. Therefore, careful clinicopathological evaluation is essential for establishing a diagnosis [3].

Human immunodeficiency virus (HIV) infection is associated with persistent immune dysregulation, despite effective antiretroviral therapy. Although IPT has occasionally been reported in people living with HIV, CNS involvement is rare [4,7,8], and multifocal CNS disease has not been described in patients with well-controlled HIV infection. Here, we report a unique case of multifocal IPT involving the orbit, skull base, and cauda equina in a patient with long-term virological suppression and review the published literature on HIV-associated IPT.

## Case presentation

A man in his 50s was diagnosed with acquired immunodeficiency syndrome (AIDS) 13 years before presentation after developing *Pneumocystis pneumonia*, esophageal candidiasis, and cytomegalovirus infection. At diagnosis, his HIV-RNA level was 224,000 copies/mL, and his CD4-positive T-cell count was 7 cells/μL. Antiretroviral therapy with darunavir/ritonavir and abacavir/lamivudine was initiated, resulting in sustained virological suppression.

Eleven years before presentation, he developed IgA nephropathy confirmed by renal biopsy and was treated with steroid pulse therapy and tonsillectomy. Progressive renal dysfunction eventually led to maintenance hemodialysis four years prior to presentation.

One year before admission, he developed headaches, although brain magnetic resonance imaging (MRI) revealed no abnormalities. In January of the index year, he noticed progressive visual impairment in his right eye. Repeat MRI revealed a soft tissue lesion adjacent to the anterior skull base with orbital involvement (Figure 1). At that time, HIV-RNA remained undetectable, and the CD4-positive cell count was approximately 500 cells/μL.

**Figure 1:**
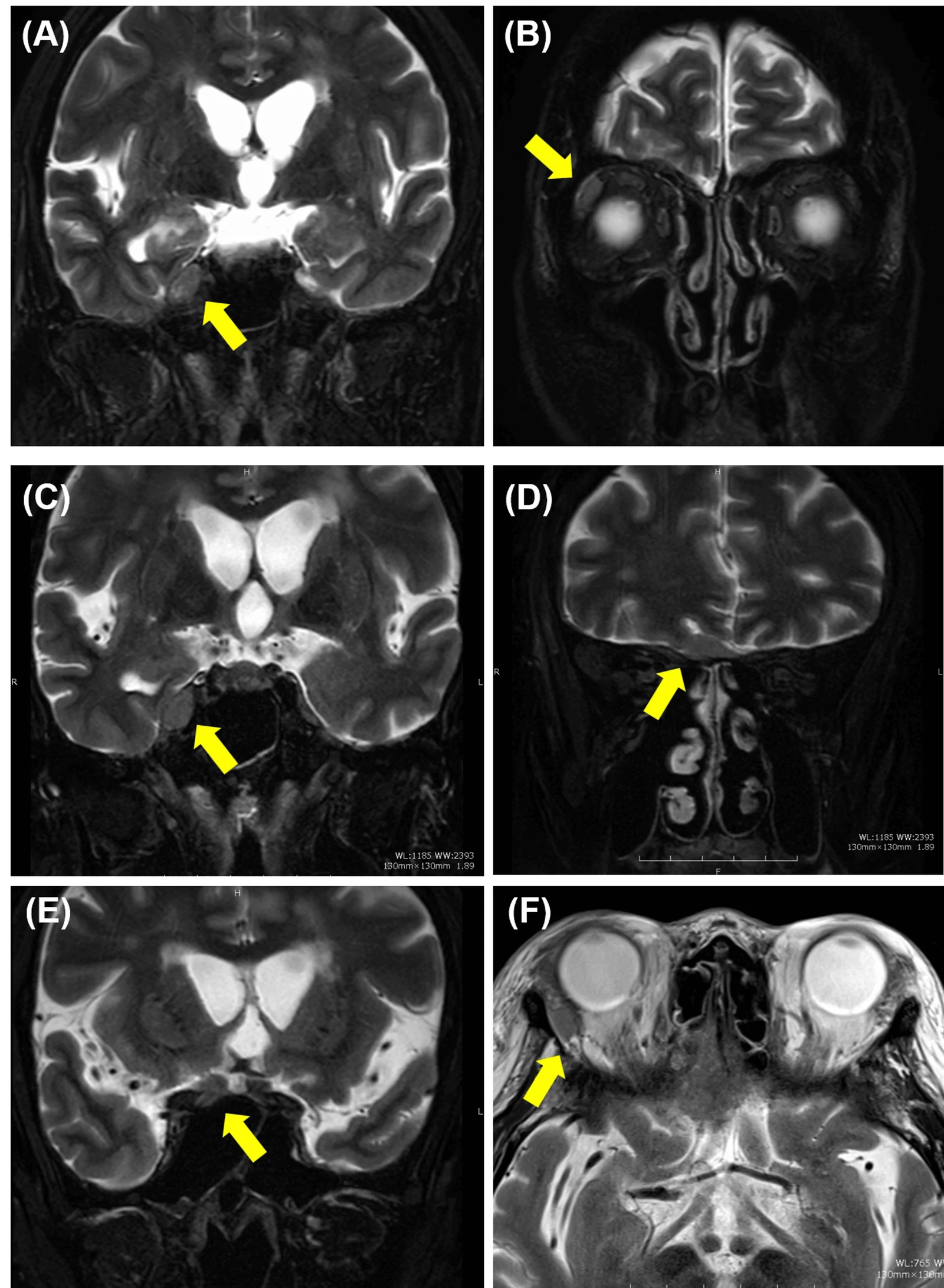
Serial brain and orbital magnetic resonance imaging (MRI) findings Coronal short tau inversion recovery (STIR) images obtained in January (A, B) and March (C-E) and an axial fluid-attenuated inversion recovery (FLAIR) image obtained in March (F) demonstrating multifocal soft tissue lesions (yellow arrows). In January, STIR images showed lesions involving the anterior skull base (A) and right lacrimal gland (B). Follow-up MRI performed in March demonstrated enlargement of the skull base lesion (C), newly developed lesions involving the suprasellar/optic chiasm region (D) and pituitary region (E), and progression of the right orbital lesion (F). These serial MRI findings were temporally associated with the patient's rapidly progressive bilateral visual deterioration and demonstrated progressive multifocal inflammatory involvement of the orbit and central nervous system.

In February, the patient developed a COVID-19 infection. Subsequently, visual acuity declined precipitously. By March, visual acuity in the right eye had declined to light perception (LP). The left eye subsequently showed a rapid decline in visual acuity from 1.2 to LP over approximately six weeks, with visual field testing demonstrating preservation of only the nasal one-quarter of the visual field (Figure 2).

**Figure 2:**
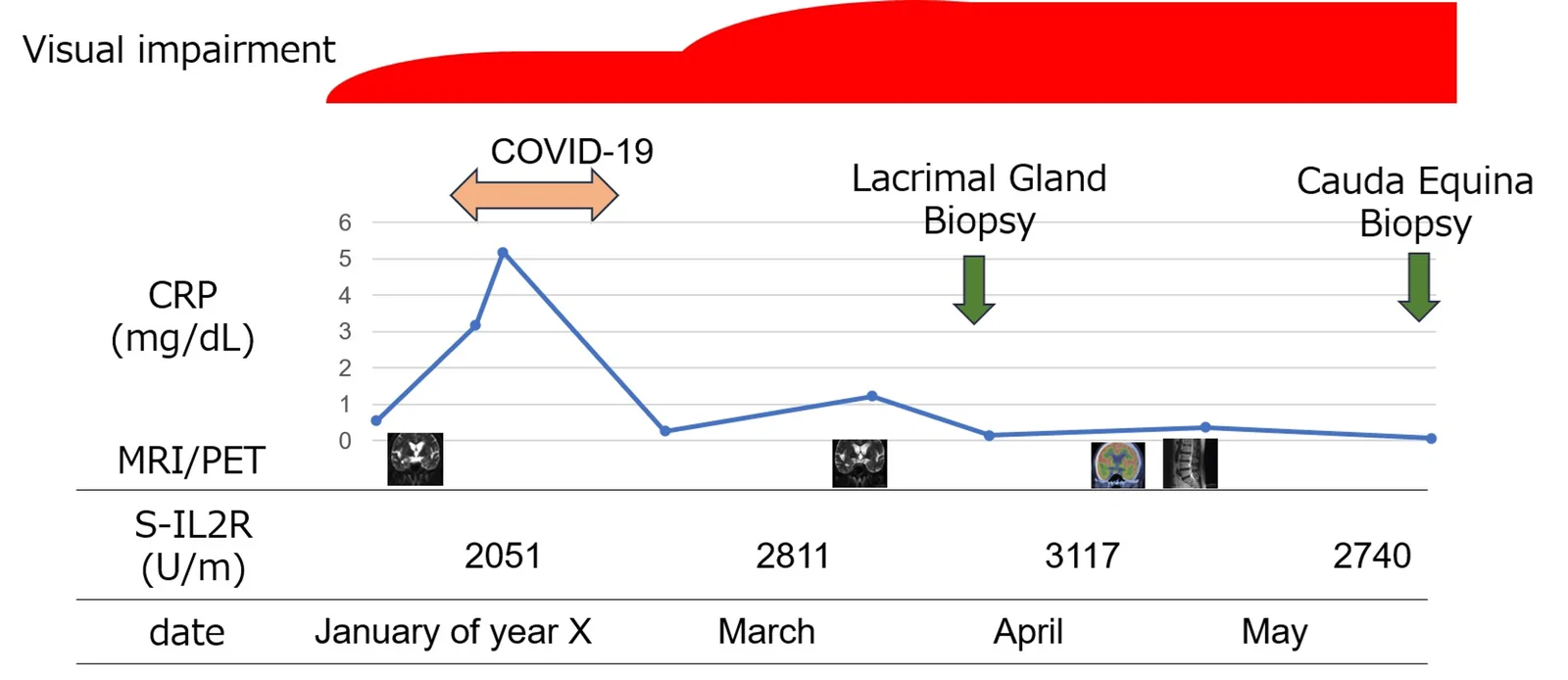
Clinical course of the patient The timeline illustrates the patient's clinical course from the onset of visual symptoms through follow-up. The blue line represents serum C-reactive protein (CRP) levels, and serum soluble interleukin-2 receptor (sIL-2R) levels are shown below the timeline. Representative MRI and FDG-PET/CT images are displayed at the corresponding time points. COVID-19 infection occurred during the early phase of the disease and was temporally associated with rapid progression of visual impairment. Lacrimal gland and cauda equina biopsies were performed in April and May, respectively, leading to the histopathological diagnosis of inflammatory pseudotumor. Despite the absence of specific treatment, CRP levels gradually normalized, and the patient's condition remained clinically stable throughout follow-up.

Laboratory investigations revealed no evidence of active opportunistic infection. HIV-RNA was undetectable, the CD4-positive cell count was 502 cells/μL, the serum IgG4 level was within the normal range, and antinuclear antibodies were negative (Table 1).

**Table 1:** Laboratory and cerebrospinal fluid findings during the diagnostic evaluation Blood samples were obtained in March and cerebrospinal fluid was collected in April of the index year. WBC: white blood cell count; Hb: hemoglobin; Plt: platelet count; Alb: albumin; T-Bil: total bilirubin; AST: aspartate aminotransferase; ALT: alanine aminotransferase; LD: Lactate dehydrogenase; Crea: creatinine; CRP: C-reactive protein; CSF: cerebrospinal fluid; TP: total protein; Glu: glucose

Parameter	Value	Unit	Reference range
WBC	7600	/µL	3,300-8,600
Eosin	10	%	0.4-8.6
Neutrophils	50	%	42.4-75.0
Lymphocytes	31	%	18.2-47.7
Monocytes	9	%	3.3-9.0
Hb	11.5	g/dL	11.6-14.8
Plt	16.8 × 10^4^	/µL	15.8-34.8 × 10^4^
Alb	4.1	g/dL	4.1-5.1
T-Bil	0.4	mg/dL	0.4-1.5
AST	14	U/L	13-30
ALT	21	U/L	10-42
LD	228	U/L	124-222
Crea	9.39	mg/dL	0.46-0.79
CRP	0.55	mg/dL	0.00-0.14
HIV-RNA	Undetectable	copies	undetectable
CD4-positive T cell	502	/uL	
IgG	1513	mg/dL	861-1747
IgA	433	mg/dL	93-393
IgM	29	mg/dL	22-183
IgG4	68.3	mg/dL	11-121
TP	274	mg/dL	8-43
Glu	57	mg/dL	50-75
Cell counts	79	/uL	
Mononuclear	97.5	%	
Polymorphonuclear	2.5		
Malignant cell	Negative		

MRI performed in March demonstrated enlargement of the skull base and orbital lesions, with additional involvement surrounding the optic chiasm, bilateral optic nerves, pituitary region, and right lacrimal gland (Figure 1). To evaluate the extent of disease, FDG-PET/CT was subsequently performed in April and demonstrated increased uptake in these lesions as well as additional uptake within the cervical spinal canal and cauda equina. Subsequent spinal MRI confirmed nodular lesions in the cervical spinal canal and cauda equina, which prompted a cauda equina biopsy for histopathological diagnosis (Figure 3).

**Figure 3:**
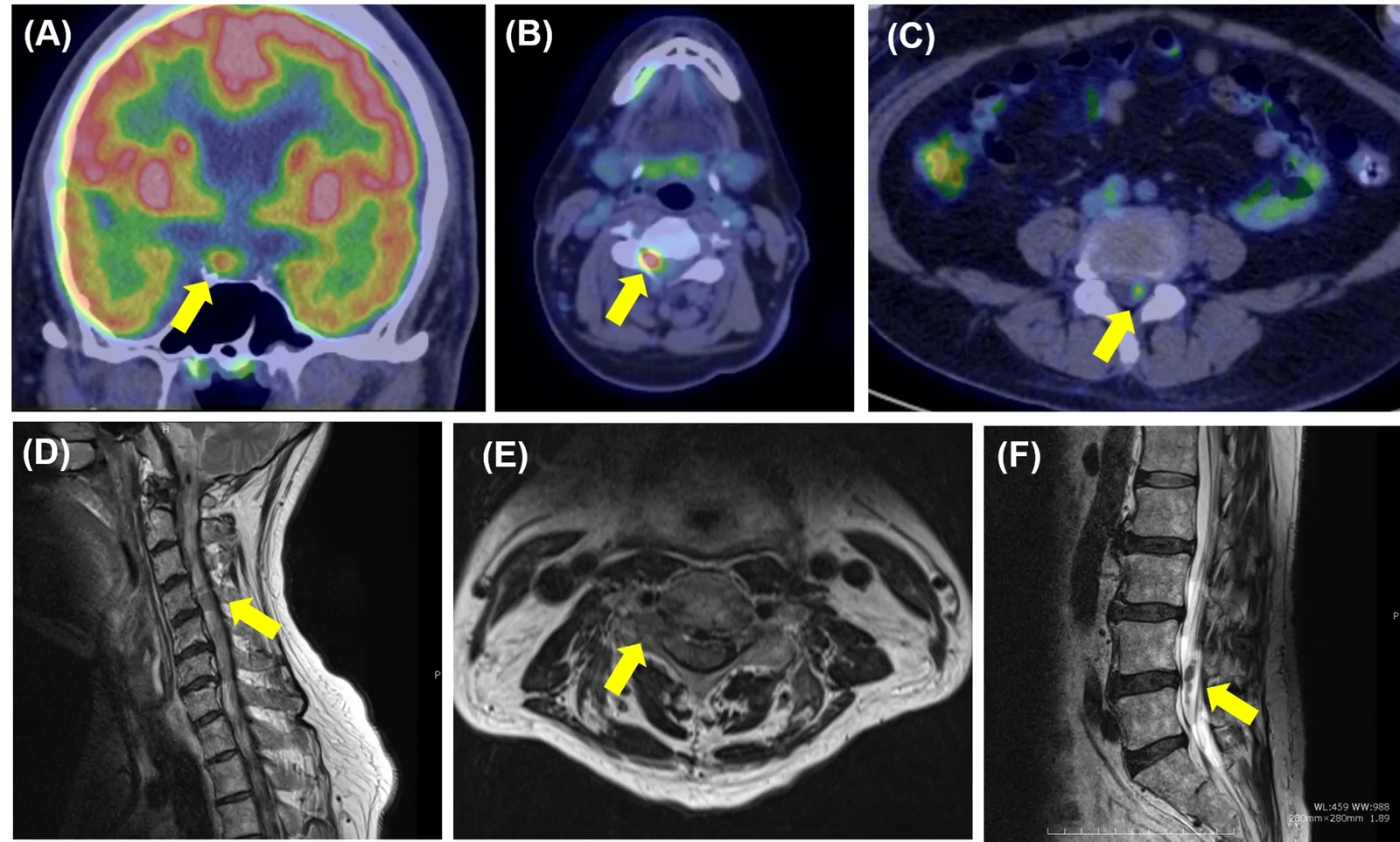
Positron emission tomography/computed tomography (PET/CT) and spinal MRI and findings Coronal (A) and axial (B, C)^18^F-fluorodeoxyglucose (FDG) PET/CT images obtained in April for systemic disease evaluation demonstrate increased FDG uptake in the pituitary region (A), cervical spinal canal (B), and cauda equina (C), indicating metabolically active inflammatory lesions. These spinal lesions identified on FDG-PET/CT prompted spinal MRI. Sagittal (D, F) and axial (E) T2-weighted MRI images subsequently confirmed multifocal soft tissue lesions (yellow arrows), including a lesion within the right cervical spinal canal at the C4/5 level (D, F) and nodular lesions involving the cauda equina (E).

Cerebrospinal fluid examination revealed lymphocytic pleocytosis (79 cells/μL, 97.5% mononuclear cells) without malignant cells. Cytological examination revealed lymphocytes and monocytes but no evidence of fungal infection, viral cytopathic changes, or malignancy. Lacrimal gland biopsy revealed infiltration by small lymphocytes with mixed CD20-positive B cells and CD3-positive T cells (Figure 4).

**Figure 4:**
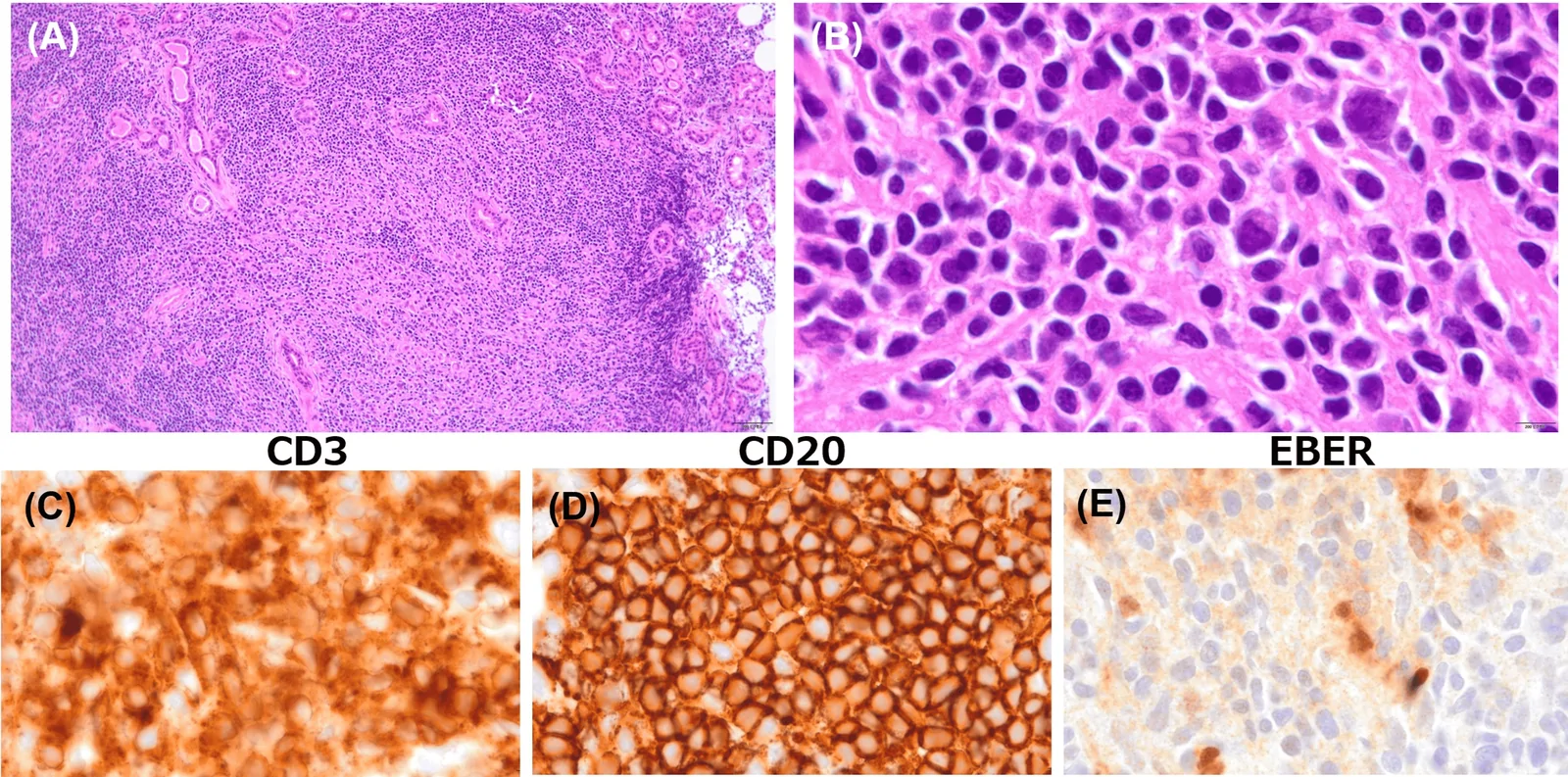
Histopathological findings of the right lacrimal gland biopsy Hematoxylin and eosin (H&E) staining demonstrates dense lymphoid infiltration replacing the lacrimal gland parenchyma. (B) H&E staining shows the infiltration of predominantly small mature lymphocytes without significant cytological atypia. Immunohistochemical staining demonstrates a mixed population of CD3-positive T cells (C) and CD20-positive B cells (D). (E) Epstein-Barr virus-encoded RNA (EBER) in situ hybridization revealed scattered EBER-positive cells. Overall, the histopathological findings were consistent with an inflammatory lesion and showed no evidence of malignant lymphoma, inflammatory myofibroblastic tumor, IgG4-related disease, or IPT-like follicular dendritic cell sarcoma. Original magnification: ×40 (A) and ×400 (B-E)

No restriction of the light chain was observed. Although scattered EBER-positive cells were observed, they were interpreted as most likely representing a reactive finding rather than a pathogenic process. In addition, CD23 immunostaining demonstrated no abnormal proliferation of follicular dendritic cells, arguing against EBV-associated IPT-like follicular dendritic cell sarcoma (Figure 5).

**Figure 5:**
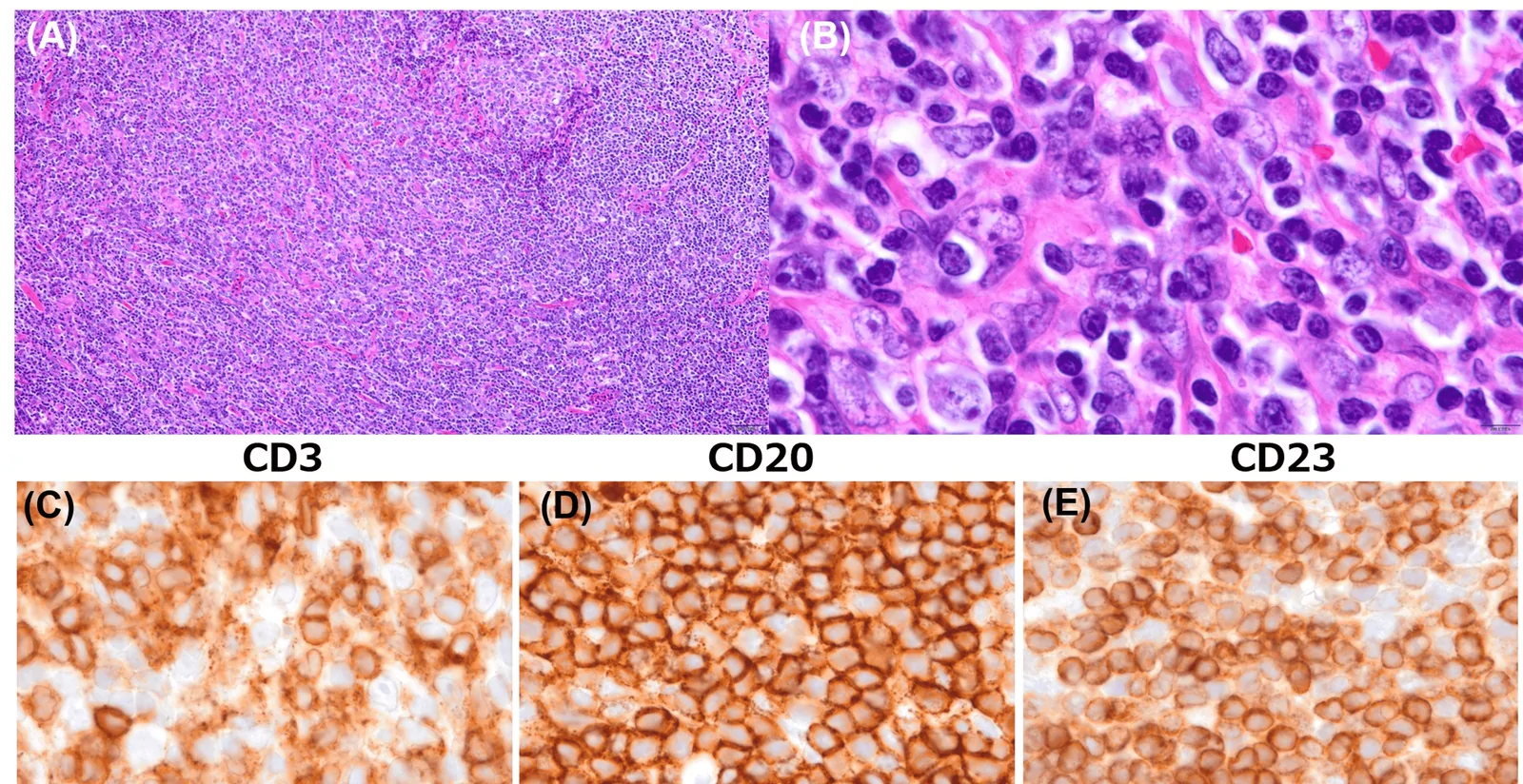
Histopathological findings of the cauda equina biopsy (A) Hematoxylin and eosin (H&E) staining (low-power view) demonstrates dense lymphoid infiltration with fibrosis. (B) High-power H&E staining shows infiltration predominantly by small mature lymphocytes without significant cytological atypia or evidence of neoplastic proliferation. Immunohistochemical staining demonstrates a mixed population of CD3-positive T cells (C) and CD20-positive B cells (D). CD23 staining (E) shows no abnormal proliferation of follicular dendritic cells. Overall, the histopathological findings were consistent with an inflammatory lesion and showed no evidence of malignant lymphoma. Original magnification: ×40 (A) and ×400 (B-E)

After multidisciplinary discussion, the lesions were diagnosed as inflammatory pseudotumor based on comprehensive clinicopathological evaluation, including microbiological investigations, which excluded malignant lymphoma, IMT, IPT-like follicular dendritic cell sarcoma, IgG4-related disease, and infectious etiologies. Because the lesions were multifocal and surgical resection was not feasible, and visual recovery was considered unlikely, conservative management was selected. During approximately 24 months of follow-up, the lesions remained generally stable. Although partial regression of some lesions and the appearance of new lesions were observed radiologically, no clinically significant neurological deterioration or new neurological deficits developed. Visual impairment, including the visual field defect, remained unchanged without recovery.

## Discussion

IPT is an uncommon inflammatory mass lesion characterized by a variable mixture of lymphocytes, plasma cells, histiocytes, and fibroblastic proliferation. Although originally described in the lung [9], IPT has subsequently been reported in numerous anatomical locations, including the orbit, liver, spleen, gastrointestinal tract, and CNS [1-3]. Because its clinical, radiological, and pathological features overlap with those of inflammatory and neoplastic disorders, IPT remains a diagnostic challenge and is generally considered a diagnosis of exclusion.

The differential diagnoses in the present case included IMT, IPT-like follicular dendritic cell sarcoma, IgG4-related disease, malignant lymphoma, and infectious diseases [2]. IMT shares several pathological features with IPT but is considered a true neoplasm characterized by myofibroblastic proliferation and, in approximately half of the cases, ALK gene rearrangement. In our patient, no convincing spindle cell proliferation suggestive of IMT was observed. Similarly, although scattered EBER-positive cells were observed, they may occasionally be seen in reactive inflammatory lesions. Furthermore, CD23 immunostaining demonstrated no abnormal proliferation of follicular dendritic cells, making EBV-associated IPT-like follicular dendritic cell sarcoma unlikely. Serum IgG4 levels were within the normal range, and the histopathological findings were inconsistent with IgG4-related disease. Furthermore, repeated pathological examinations failed to reveal the presence of lymphoma or infectious organisms. Collectively, these findings support the diagnosis of IPT.

To better characterize IPT in people living with HIV, we performed a literature search. A literature search was performed in PubMed for articles published from January 2000 to June 2026 using the keywords "HIV" AND "inflammatory pseudotumor." Publications not involving HIV-infected patients, review articles, and cases subsequently diagnosed with distinct clinicopathological entities (e.g., IgG4-related disease) were excluded. Finally, a total of 15 cases of IPT or IPT-like lesions associated with HIV infection were identified (Table 2) [4,6-8,10-19]. Among these 15 cases, orbital involvement was reported in two cases, both of which were diagnosed with IPT [7,8]. The two previously reported HIV-associated cases with CNS involvement differed substantially from the present case. One represented a mycobacterial spindle cell pseudotumor involving the spinal cord in a patient with newly diagnosed HIV infection and a CD4 count of 15 cells/μL, while the other was a JCV-associated IPT of the brain in a patient with a CD4 count of 96 cells/μL [4,5]. In contrast, our patient had sustained virological suppression with a preserved CD4-positive T-cell count (502 cells/μL), and extensive investigations revealed no evidence of an opportunistic infection. These findings suggest that multifocal CNS IPT may occur even in the setting of effective immune reconstitution and should not be regarded solely as a manifestation of advanced immunodeficiency. Furthermore, all previously reported lesions were confined to a single anatomical site, and no case demonstrated simultaneous multifocal involvement of the CNS. To the best of our knowledge, no previous report has described HIV-associated IPT with simultaneous multifocal CNS involvement.

**Table 2:** Reported cases of inflammatory pseudotumor in patients with HIV infection identified through a PubMed literature search (2000-2026) Cases were identified by a PubMed search using the keywords "HIV" AND "inflammatory pseudotumor" for articles published between January 2000 and June 2026. Review articles, duplicate reports, cases without confirmed HIV infection, and cases subsequently diagnosed as distinct clinicopathological entities (e.g., IgG4-related disease and inflammatory myofibroblastic tumor) were excluded. Infection-associated inflammatory pseudotumorous lesions (e.g., mycobacterial spindle cell pseudotumor and virus-associated IPT-like lesions) were retained because they represent part of the reported spectrum of HIV-associated inflammatory pseudotumorous lesions. ref: reference, CNS: central nervous system, IPT: inflammatory pseudotumor, MCPS: mycobacterial spindle cell pseudotumor, ART: antiretroviral therapy, JCV: John Cunningham virus, CMV: cytomegalovirus, HSV: herpes simplex virus, NR: not reported

First author	publication year	Age/gender	HIV status	CD4	Organ involved	Final diagnosis	Treatment	Outcome
Cusimano et al. [10]	2025	30/M	Suppressed	96	Pancreas	MSCP	Antimycobacterial therapy	Improved
Aminparast et al. [11]	2025	18/F	Suppressed	NR	Epigastric region	MSCP	Antimycobacterial therapy	Improved
Duong et al. [5]	2024	50s/M	Newly diagnosed	15	Spinal cord	MSCP	Antimycobacterial therapy	Improved
Bai et al. [12]	2020	52/M	NR	NR	Rectal	HSV-associated IPT	Antiviral therapy	Improved
Gore et al. [8]	2017	56/M	NR	NR	Orbit	IPT+chronic sinusitis	Antibiotics+steroid	Improved
Mozzo et al. [7]	2016	NR/F	Suppressed	NR	Orbit	IPT	Observation	Improved
Charles P et al. [13]	2016	44/F	Suppressed	343	Rectal	HSV-associated IPT	Antiviral therapy+thalidomide	Improved
Ramotar et al. [13]	2016	49/F	Suppressed	500	Cervical lymph node	IPT	Surgical resection	Improved
Minami et al. [6]	2013	59/M	Newly diagnosed	49	Duodenum	CMV-associated IPT	Antiviral therapy	Improved
Gonzalez-Duarte et al. [4]	2009	37/F	Suppressed	96	Brain	JCV-associated IPT	Observation	Stable
Mazhari et al. [15]	2006	27/M	NR	NR	Testis	IPT	Surgical resection	Improved
Navai et al. [16]	2005	30/M	Newly diagnosed	385	Testis	IPT	Surgical resection	Improved
Braun et al. [17]	2003	38/F	Untreated	91	Spleen	IPT	Surgical resection	Improved
Vaideeswar et al. [18]	2000	32/M	Newly diagnosed	NR	Cervical lymph node	IPT	Surgical resection	Stable
Aboulafia et al. [19]	2000	43/M	Suppressed	185	Duodenum	IPT	Thalidomide	Improved
Present case	2026	50s/M	Suppressed	502	Orbit, Brain, Spinal cord	IPT	Observation	Stable

Moreover, multifocal CNS IPT is extremely rare even in patients without HIV infection [1]. Most reported intracranial IPTs present as solitary dural-based masses that radiologically mimic meningiomas, whereas simultaneous involvement of multiple intracranial and spinal sites has rarely been described. Therefore, the extensive distribution observed in our patient appears to represent an exceptionally uncommon manifestation, regardless of HIV status.

The present case is notable for several reasons. First, the patient developed multifocal lesions involving the orbit, skull base, optic nerve sheaths, optic chiasm, pituitary region, and cauda equina, resulting in rapidly progressive bilateral visual impairment. Second, these lesions occurred despite long-term virological suppression with antiretroviral therapy and sustained immune reconstitution, with CD4-positive T-cell counts consistently exceeding 400 cells/μL. These findings suggest that persistent immune dysregulation despite successful immune reconstitution, rather than profound immunodeficiency, may contribute to IPT development in people living with HIV.

Another intriguing aspect is the temporal relationship between COVID-19 infection and rapid disease progression. MRI performed before COVID-19 infection had already demonstrated orbital and skull base lesions, and visual impairment worsened markedly after COVID-19. Although causality cannot be established from a single case, COVID-19-induced immune activation may accelerate the progression of pre-existing inflammatory lesions.

The optimal treatment strategy for IPT depends on the disease location, extent, and underlying etiology. Surgical resection is generally preferred for localized lesions [3], whereas corticosteroids, immunosuppressive agents, radiotherapy, and treatment of the underlying cause have been used for multifocal or unresectable disease [2,8]. In the present case, complete surgical resection was not feasible because of the extensive involvement of the CNS, and irreversible visual loss had already occurred by the time of diagnosis. Although corticosteroid therapy was considered, conservative management was ultimately selected following a multidisciplinary discussion because visual recovery was considered unlikely, and serial imaging demonstrated relative radiological stability with partial regression of some lesions. During follow-up, the lesions remained largely stable without clinically significant progression, further supporting their non-neoplastic nature.

This case has several limitations. Histopathological confirmation was obtained from the lacrimal gland and cauda equina, but not from intracranial lesions. In addition, molecular analyses such as ALK immunohistochemistry or gene rearrangement studies were not performed; therefore, IMT cannot be excluded with absolute certainty. Finally, the contributions of HIV and COVID-19 to disease pathogenesis remain speculative.

## Conclusions

We report a unique case of a multifocal inflammatory pseudotumor involving the orbit, skull base, optic apparatus, and cauda equina in a patient with a well-controlled HIV infection. Our review of the literature indicates that this pattern of extensive multifocal CNS involvement has not previously been reported in people living with HIV. This case expands the recognized clinical spectrum of HIV-associated IPT and underscores that IPT should be considered in the differential diagnosis of multifocal inflammatory CNS mass lesions, even in patients with sustained virological suppression and immune reconstitution.
